# Sublingual Nucleotides Prolong Run Time to Exhaustion in Young Physically Active Men

**DOI:** 10.3390/nu5114776

**Published:** 2013-11-21

**Authors:** Sergej M. Ostojic, Kemal Idrizovic, Marko D. Stojanovic

**Affiliations:** Center for Health, Exercise and Sport Sciences, Deligradska 27, Stari DIF, Belgrade 11000, Serbia; E-Mails: kemal.idrizovic@chess.edu.rs (K.I.); marko.stojanovic@chess.edu.rs (M.D.S.)

**Keywords:** nucleotides, immunoglobulins, endurance, fatigue, exercise

## Abstract

Although dietary nucleotides have been determined to be required for normal immune function, there is limited direct interventional evidence confirming performance-enhancing effects of sublingual nucleotides in humans. A double-blind, placebo-controlled, randomized trial was conducted to evaluate the effect of sublingual nucleotides (50 mg/day) administered for 14 days in thirty young healthy physically active males, on endurance performance and immune responses. Fasting white blood cell count, natural killer cells (NKC) number, NKC cytotoxic activity, and serum immunoglobulin (IgA, IgM, IgG), and time to exhaustion, peak rate of perceived exertion, peak heart rate, and peak running speed during the exercise test were measured at baseline (day 0) and post-intervention (day 14). Time to exhaustion, as well as serum immunoglobulin A and NKC cytotoxic activity, were significantly higher at day 14 (*p* < 0.05) in participants supplemented with nucleotides compared with those who consumed placebo. No significant differences in other parameters were observed between groups at post-intervention. No volunteers withdrew before the end of the study nor reported any vexatious side effects of supplementation. The results of the present study suggest that sublingual nucleotides may provide pertinent benefit as both an ergogenic and immunostimulatory additive in active males.

## 1. Introduction

Nucleotides are a group of organic molecules made up of a nitrogenous base, a five-carbon sugar, and a phosphate group. The main biological role of nucleotides is to form the building blocks of nucleic acids, such as DNA and RNA. Aside from acting as precursors of nucleic acids, nucleotides also participate in cellular signaling and metabolism, serving as convenient and universal carriers of metabolic energy and high-energy electrons [1]. Nucleotides are substances that are synthesized endogenously—they have important effects on the growth and development of cells that have a rapid turnover, such as those in the immune or gastrointestinal system [1,2]. However, under certain circumstances (e.g., deficient diet, heavy exercise-related suppression of immune parameters, recovery) exogenous nucleotides may be semi-essential, optimizing the function of the immune system and energy utilization when the endogenous supply may limit the synthesis of nucleotides [3,4]. However, there has been only limited scientific research regarding the true biological activity of exogenously administered nucleotides in humans, particularly in the athletic environment.

Several clinical studies have reported beneficial effects of nucleotide supplementation on gut microflora, diarrhea, lipid metabolism and immune function in formula-fed infants [5,6,7,8]. Short-term oral supplementation with nucleotides in physically active males may blunt the response of the hormones associated with physiological stress [9], and boost immune responses to short term high intensity cycling [10]. Although the exact mechanism of action of nucleotides is currently unknown, nucleotides can mitigate stress on the body induced by endurance exercise and provide added nutritional support for strengthening the immune system. It seems that athletes, during high-intensity endurance training and post-exercise recovery, would benefit the most from using nucleotides. However, utilization of dietary nucleotides has been hampered by low bioavailability (<10%) following oral administration [11]. Several studies researched the absorption process of nucleotides and the need for modification of nucleotides for better absorption [12,13]. To avoid the degradation of nucleotides in the gastrointestinal tract and first pass metabolism in the liver after oral intake [14], sublingual administration of nucleotides may be a more advantageous route of application. A pilot study from our group [15] reported affirmative effects of sublingual nucleotides on post-exercise drop of salivary and serum immunoglobulins. However, no studies so far examined the performance-enhancing effects of sublingual nucleotides in humans. Therefore, the main aims of this study were to investigate whether daily sublingual administration of nucleotides affected endurance exercise and concomitant immune responses in healthy active men, and to determine how many participants experienced adverse effects at follow-up after this treatment. In the current study we tested the hypothesis that daily sublingual administration of 50 mg of nucleotides formulation for 14 days would augment exercise performance and positively affect serum immunoglobulins at post-intervention, but not increase prevalence of adverse effects as compared to placebo in physically active males.

## 2. Methods

### 2.1. Participants

Double-blind, placebo-controlled, randomized trial was conducted to assess the effect of sublingual nucleotides (50 mg daily divided over three portions to be taken at regular intervals throughout the day) as compared to placebo, both administered for 14 days in healthy young male participants. Since no dose-response studies are available, dosage of nucleotides administered was used as a dose known to effectively increase salivary and serum immunoglobulins after 2 weeks of administration [15]. Furthermore, the estimated daily turnover for nucleotides is about 450 to 750 mg per day in adult humans [16] with up to 15% of the total daily requirement was calculated to come from dietary sources, and 50 mg are close to this daily needs. This study was conducted according to the guidelines laid down in the Declaration of Helsinki and all procedures involving human subjects were approved by the Center’s Ethical Review Board (Approval No. 175-03/10). Written informed consent was obtained from all participants. Data were obtained from university students who were regularly engaged in aerobic exercise five times per week during the past 2 years or more, and who were between 20 and 25 years of age. Potential candidates were not included in the study if any of the following criteria were present: (1) a history of metabolic disease; (2) musculoskeletal dysfunction; (3) known heart disease; (4) smoking; (5) use of any performance-enhancing drugs or dietary supplements within the past 30 days; (6) an impaired response to exercise test; and (7) residence outside the city of Belgrade, or unwillingness to return for follow-up. Following recruitment, thirty participants (*n* = 30) met the criteria to participate in the study. Participants were asked to maintain their usual dietary intake and not to change their physical activity patterns during the study. Additional dietary analysis of nucleotides ingested during the study was not administered since no relevant data are available for calculation of nucleotides content in different foods. Participants were randomly assigned to receive nucleotides (*n* = 15) or placebo (*n* = 15), and were instrumented for blood sampling and endurance running test at the start (day 0) and at the end of the intervention period (day 14). Randomization was made sequentially by an independent pharmacist, who dispensed the intervention, but was not otherwise involved in the collection of data. Two groups (nucleotides *vs*. placebo) were homogenous for age, height, body mass index, body fat, and maximal oxygen uptake (Table 1).

**Table 1:** Characteristics of the participants (mean ± SD).

Variables	Nucleotides (*n* = 15)	Placebo (*n* = 15)
Age (years)	22.6 ± 2.1	21.9 ± 1.8
Weight (kg)	78.9 ± 8.1	80.2 ± 6.5
Height (cm)	180.5 ± 4.3	182.1 ± 6.3
Body mass index (kg/m^2^)	24.2 ± 1.6	24.2 ± 2.0
Body fat (%)	11.5 ± 2.7	10.8 ± 4.0
Maximal oxygen uptake (mL/kg/min)	50.4 ± 5.9	49.3 ± 6.6

### 2.2. Intervention

Nucleotides formulation contained cytidine 5′-monophosphate, uridine 5′-monophosphate, guanosine 5′-mono-phosphate and adenosine 5′-mono-phosphate and was extracted and partially purified (90%) from germinated barley seeds during sporulation. Barley was used as a source of nucleotides due to its rich nucleotide sequence diversity [17], and high cyclic nucleotide phosphodiesterase activity [18]. The optimized procedure involved extraction with a monophasic mixture of methanol, chloroform, formic acid and water, and purification of the nucleotide extract by a batch treatment with poly-*N*-vinylpyrrolidone [19]. Nucleotide profile of the formulation used for the present study corresponds to the infant milk formula fortified with nucleotides [20]. Both nucleotides and placebo were provided in spray device for sublingual administration, with placebo (tap water) was similar in appearance, volume and taste. Appearance and taste were standardized by addition of citric acid and fructose to be undistinguishable from each other.

### 2.3. Experimental Procedure

Participants visited the laboratory on two occasions: upon the initial intervention and 14 days following the intervention. In the 24 h before the measurement, the participants did not participate in any prolonged exercise or drinking alcoholic and/or caffeine beverages. All measurements were taken between 9 and 11 a.m. after an overnight fast of between 10 and 12 h. Venous blood samples were collected from a radial vein with white blood cell (WBC) count, natural killer cells (NKC) number, NKC cytotoxic activity and serum immunoglobulins (IgA, IgM, IgG) concentration determined. WBC count was determined using a Coulter blood counter (Model S-plus II, Coulter Electronics Inc., Hialeah, FL, USA). NKC number was determined by flow cytometry (Becton Dickinson Immunocytometry Systems, San Jose, CA, USA), with NKC cytotoxic activity determined using NKTEST Kit (ORPEGEN Pharma, Heidelberg, Germany). Serum immunoglobulins (IgG, IgA and IgM) levels were determined by a commercial nephelometry assay using a BN-II device (Dade Behring, Marburg, Germany). After the blood sample was taken, standardized snack (~1046 kJ) and 250 mL of tap water were given to participants 30 min before the exercise test.

In an effort to eliminate anticipated test-retest variability and establish reliability of treadmill running protocols, four familiarization sessions were conducted over a 4-week period before day 0. The sessions consisted of repeated practicing of treadmill protocol. The familiarization sessions involved teaching specific exercise techniques for treadmill running and allowing submaximal practice. Maximal practice took place 2 times in order to reduce any learning effects and allow for stability of the data. By the fourth familiarization session, intraclass R was above 0.98 for treadmill test, indicating the elimination of the test-retest variability for the participants. Exercise test was performed according to incremental protocol using a treadmill system (Trackmaster TMX425C, Newton, KS, USA). The running protocol consisted of 1-min workloads with participants beginning at a running speed of 8 km/h and increased by 2 km/h for each of subsequent workloads until volitional exhaustion. Time to exhaustion, peak rate of perceived exertion by Borg’s 6–20 scale [21], and peak heart rate (Polar S810, Polar Electro, Kempele, Finland) during the test were noted. Participants were instructed to report any adverse effects of administration through open-ended questionnaire.

### 2.4. Statistical Analyses

The primary efficacy outcome was the change in time to exhaustion at 2 weeks after administration. Prior to statistical analysis, all continuous data were assessed for normal distribution using a Kolmogorov-Smirnov test. Statistical significance was assessed using Students’s *t* test for correlated samples. Two-way analysis of variance with repeated measures was used to establish if any significant differences existed between participants’ responses over time of intervention (0 *vs*. 2 weeks). Where significant differences were found, the Tukey test was employed to identify the differences at post-intervention. Data are presented as means with their standard deviations in all statistical analyses; *P* values < 0.05 were considered significant.

## 3. Results

No significant differences in baseline data were found between the intervention groups at day 0. Changes in fasting serum immunological profiles and exercise performance indices during the study are presented in Table 2. Results indicated significant treatment *vs*. time interaction for serum immunoglobulin A, NKC cytotoxic activity, and time to exhaustion during the study (*p* < 0.05). No significant differences in other parameters were observed between groups at post-intervention.

**Table 2:** Immunological and exercise performance variables in nucleotides and placebo trials.

Variables	Nucleotides (*n* = 15)		Placebo (*n* = 15)	
	Pre	Post	Pre	Post
WBC count (×10^9^/L)	6.2 ± 0.9	6.3 ± 0.6	6.5 ± 1.5	6.5 ± 1.4
NKC count (number cell/mm^3^)	299.2 ± 77.3	306.1 ± 85.3	274.6 ± 64.3	275.9 ± 102.0
NKC cytotoxic activity (LU)	35.5 ± 12.3	48.3 ± 10.2 *^,†^	29.9 ± 9.8	29.5 ± 16.3
Immunoglobulin A (mg/dL)	205.1 ± 38.9	212.3 ± 29.7 ^†^	198.4 ± 41.4	194.2 ± 14.3
Immunoglobulin M (mg/dL)	99.4 ± 14.1	101.4 ± 25.0	96.8 ± 27.8	97.0 ± 18.9
Immunoglobulin G (mg/dL)	1430.5 ± 212.0	1440.5 ± 493.6	1299.1 ± 310.9	1292.6 ± 671.2
Time to exhaustion (s)	315.4 ± 20.8	330.5 ± 18.6 *^,†^	303.7 ± 17.2	306.1 ± 25.8
Peak RPE (score)	18.9 ± 1.4	19.5 ± 1.6	19.3 ± 0.7	19.7 ± 1.0
Peak heart rate (beats per min)	195 ± 3	195 ± 6	192 ± 5	194 ± 3
Peak treadmill speed (km/h)	17.8 ± 1.4	18.0 ± 0.9	18.2 ± 1.2	18.2 ± 2.3

* Indicates significant difference pre- *vs*. post at *p* < 0.05; ^†^ significant difference nucleotides *vs*. placebo at *p* < 0.05. Values are means ± SD. WBC—white blood cells; NKC—natural killer cells; RPE—rates of perceived exertion.

NKC cytotoxic activity increased significantly from before to after administration in nucleotides-administered participants (35.5 ± 12.3 LU *vs*. 48.3 ± 10.2 LU; 95% confidence interval [CI] 4.4–21.3, *p* = 0.005). Time to exhaustion was significantly improved in nucleotides group after the administration period (315.4 ± 20.8 s *vs*. 330.5 ± 18.6 s; 95% CI: 0.3–29.9, *p* = 0.04). There were no significant differences in serum immunological outcomes and exercise performance from before to after administration in the placebo group. No volunteers withdrew before the end of the study nor reported any side effects from the supplementation.

## 4. Discussion

The present study shows a significant increase in serum immunoglobulin A and NKC activity in active males who sublingually consumed daily supplements of 50 mg of nucleotides for 2 weeks; none of the participants who completed the study reported any side-effects. The roughly 5% increase in time to exhaustion during running test indicates performance-enhancing capacity of exogenous nucleotides. Our study suggests that the immunostimulatory potential of sublingual nucleotides in healthy subjects is superior as compared to oral intervention, since oral nucleotides raised serum immunoglobulin A by up to 3% [22], while bioavailability after oral nucleotides administration was less than 10% [11]. These results suggest that immune function as well as endurance performance in physically active individuals is sensitive to sublingual nucleotides.

### 4.1. Immunostimulatory Effect of Nucleotides Procedure

The first reported use of supplemental nucleotides in clinical medicine dates back about 35 years. Pita *et al*. [23] exposed preterm infants to dietary nucleotides formulation at a level similar to that found in human milk. Authors suggested that nucleotide supplementation of diet resulted in a better adaptation of milk formulas during early life through affirmative changes in the fatty acids conversion. Since then, the effects of dietary nucleotides have been extensively studied and documented for a plethora of human diseases [24]. However, only a few human studies evaluated modulation of the immune response mediated by dietary nucleotides. Exogenous nucleotides have been reported to be beneficial, especially in infants when the nutrition supply was inadequate, since they positively affect immunity and tissue growth, development and repair [25]. NKC activity and production of interleukin-2 were significantly higher in infants fed with added nucleotides at a level of 32 mg/L as compared to the unsupplemented group [26]. Navaro and co-workers [22] reported higher plasma levels of immunoglobulin M in preterm infants fed with nucleotides-supplemented milk formula from 10 days to 3 months. The authors concluded that dietary nucleotides appear to exert actions on immature human neonate lymphocytes enhancing the *in vivo* production of immunoglobulin, which may have a role in the defense capacity of neonates. A randomized controlled trial [6] showed modest improvement in antibody response in infants supplemented with formula fortified with nucleotides at 33.5 mg/L. In two studies by Mc Naughton and co-workers [9,10] the authors reported elevated salivary immunoglobulin A in a group of physically active males supplemented with nucleotides for 60 days. Ostojic and co-workers [15] found significantly increased immunoglobulin A and salivary lactoferrin (protein with bactericidal and iron-binding properties) in nucleotides-administered to active participants. In accordance with previous research, the present study shows a significant increase in serum immunoglobulin A and NKC activity in males sublingually supplemented with 50 mg of nucleotides for two weeks. This implies that nucleotides are absorbed from the mucous membrane under the tongue, enters the circulation and are available for lymphocyte subpopulation activation, and modulation of immunoglobulin production [15]. The precise mechanism of the effects of dietary nucleotides on cellular immunity is not clear. Gill [25] suggested that the exogenous nucleotides may initiate the initial phase of antigen processing and lymphocyte proliferation, modulate T-helper cell-mediated antibody production, or mediate signal membrane transduction and expression of a number of genes, some of which can directly affect the levels of cell-signaling protein molecules. Further studies are needed to explicate the mechanism of immunostimulatory effects of sublingual nucleotides, with longer administration protocol and a higher dosage of the formulation, along with proven bioavailability coupled with monitoring other indicators of immunity. Although the sample size in the present study was small, we conclude that sublingual nucleotides may contribute to improved immunity when administered sublingually in healthy active males. In practical terms, short-term sublingual nucleotides supplementation may be beneficial in preventing exercise-induced immunodepression and reducing the susceptibility to infection, and in the longer term, less inactivity and hindered exercise performance during illness in highly physically active people.

### 4.2. Nucleotides for Exercise Performance

Exogenous nucleotides may optimize tissue function when the endogenous supply limits the synthesis of nucleic acids, or utilization of nucleotides is augmented [2]. Seminal work by Broberg and Sahlin [27] reported nucleotides degradation in human skeletal muscle during prolonged exercise; thus implying increased requirement for nucleotides in physically active individuals. Dietary nucleotides have been found to help athletes by reducing the release of stress related hormones and metabolites after exercise [9], and by maintaining a higher level of antibodies, which enables the immune system to work more effectively [10]. However, the authors know of no studies that have examined the effects of exogenous nucleotides on performance outcomes during endurance exercise. Previous animal studies suggested possible effects of dietary nucleotides that could be beneficial for endurance performance, such as increased liver glycogen [28], improved fatty acids metabolism [29], augmented iron utilization [30], and/or adequate peripheral oxygen delivery [31]. In particular, neonatal rats fed with nucleotide-supplemented formula demonstrated increased erythrocyte 2,3-diphosphoglycerate concentrations [32], which could facilitate release of oxygen by decreasing the oxygen affinity of hemoglobin. Since the endurance performance is partly limited by the ability of exercising muscles to extract and utilize oxygen [33], it seems that supplementary nucleotides could intensify the peripheral oxygen delivery and subsequently the capacity for endurance exercise. The present study suggests that a nucleotide supplement, given sublingually, may significantly delay total time to exhaustion by up to 5%. Seeing that no oxygen consumption has been evaluated, amplified time to exhaustion during endurance running could be due to enhanced peripheral transportation of oxygen or some other unknown effect of sublingual nucleotides. Future research should confirm these findings by monitoring oxygen uptake within a clinical environment and elucidate the physiological mechanism of a performance-enhancing effect of sublingual nucleotides. Due to the linear relationship between heart rate (also RPE) and oxygen uptake, no differences found in heart rate and RPE at a constant exercise intensity load (treadmill speed) for the re-test probably reflects no major changes in oxygen uptake after intervention. Furthermore, other factors than the supplement that may influence the change in exercise time to exhaustion should be noted, such as hyperthermia-induced reduction of peripheral muscle activation due to decreased central activation (brain fatigue), hydration level, peripheral effect of hyperthermia on muscle fatigue, depletion of energy stores, electrolyte imbalance, and/or other factors [34], which requires more investigation. According to the results of the present study, sublingual nucleotides may be recommended as an ergogenic agent for those physically active who are aiming to keep up with highly demanding physical activities, or to enhance recovery after endurance exercise. Although long-term studies are not available at the moment, nucleotides formulation is considered relatively safe when a dose of 50 mg per day is taken for 14 days.

## 5. Conclusions

In conclusion, sublingual administration of nucleotides formulation for 14 days improved some of the immunological and exercise performance indices, above baseline and placebo level, in healthy male volunteers, with no adverse effects reported. Evidence confirmed previous animal studies [35] suggesting that sublingual nucleotides may provide some benefits as immunostimulatory and ergogenic agents. Further studies are needed to extend these results, seeking to clarify the mechanism by which improvements occur.

## References

[1] Grimble G.K., Westwood O.M. (2001). Nucleotides as immunomodulators in clinical nutrition. Curr. Opin. Clin. Nutr. Metab. Care.

[2] Dancey C.P., Attree E.A., Brown K.F. (2006). Nucleotide supplementation: A randomized double-blind placebo controlled trial of IntestAidIB in people with irritable bowel syndrome. Nutr. J..

[3] Maldonado J., Navorro J., Narbona E., Gil A. (2001). The influence of dietary nucleotides on humoral and cell immunity in the neonate and lactating infant. Early Hum. Dev..

[4] Pendergast D.R., Meksawan K., Limprasertkul A., Fisher N.M. (2011). Influence of exercise on nutritional requirements. Eur. J. Appl. Physiol..

[5] Yu V.Y. (2002). Scientific rationale and benefits of nucleotidesupplementation of infant formula. J. Paediatr. Child Health.

[6] Hawkes J.S., Gibson R.A., Roberton D., Makrides M. (2006). Effect of dietary nucleotide supplementation on growth and immune function in term infants: A randomized controlled trial. Eur. J. Clin. Nutr..

[7] Santora R., Kozar R.A. (2010). Molecular mechanisms of pharmaconutrients. J. Surg. Res..

[8] Singhal A., Kennedy K., Lanigan J., Clough H., Jenkins W., Elias-Jones A., Stephenson T., Dudek P., Lucas A. (2010). Dietary nucleotides and early growth in formula-fed infants: A randomized controlled trial. Pediatrics.

[9] Mc Naughton L., Bentley D., Koeppel P. (2007). The effects of a nucleotidesupplement on the immune and metabolic response to short term, high intensity exercise performance in trained male subjects. J. Sports Med. Phys. Fitness.

[10] Mc Naughton L., Bentley D.J., Koeppel P. (2006). The effects of a nucleotide supplement on salivary IgA and cortisol after moderate endurance exercise. J. Sports Med. Phys. Fitness.

[11] Coolen E.J., Arts I.C., Bekers O., Vervaet C., Bast A., Dagnelie P.C. (2011). Oral bioavaiability of ATP after prolonged administration. Br. J. Nutr..

[12] Li F., Maag H., Afredson T. (2008). Prodrugs of nucleoside analogues for improved oral absorption and tissue targeting. J. Pharm. Sci..

[13] Peterson L.W., McKenna C.E. (2009). Prodrug approaches to improving the oral absorption of antiviral nucleotide analogues. Expert Opin. Drug. Deliv..

[14] Bierau J., van Kuilenburg A.B.P., Gennip A.H. (2005). Nucleotides Degradation. eLS.

[15] Ostojic S.M., Obrenovic M. (2012). Sublingual nucleotides and immune response to exercise. J. Int. Soc. Sports Nutr..

[16] Smith L.H. (1973). Pyrimidine metabolism in men. N. Engl. J. Med..

[17] Cummings M.P., Clegg M.T. (1998). Nucleotide sequence diversity at the alcohol dehydrogenase 1 locus in wild barley (*Hordeum vulgare* ssp. spontaneum): An evaulation of the background selection hypothesis. Proc. Natl. Acad. Sci. USA.

[18] Vandepeute J., Huffaker R.C., Alvarez R. (1973). Cyclic nucleotide phosphodiesterase activity in barley seeds. Plant Physiol..

[19] Brown E.G., Davies D. (1989). Extraction, pre-high-performance liquid chromatographic purification, and high-performance liquid chromatographic analysis of plant nucleotides. Anal. Biochem..

[20] Pickering L.K., Granoff D.M., Erickson J.R., Masor M.L., Cordle C.T., Schaller J.P., Winship T.R., Paule C.L., Hilty M.D. (1998). Modulation of the immune system by human milk and infant formula containing nucleotides. Pediatrics.

[21] Borg G. (1998). Borg’s Perceived Exertion and Pain Scales.

[22] Navarro J., Maldonado J., Narbona E., Ruiz-Bravo A., Garcia Salmeron J.L., Molina J.A., Gil A. (1999). Influence of dietarynucleotides on plasmaimmunoglobulinlevels and lymphocyte subsets of preterm infants. Biofactors.

[23] Pita M.L., Fernandez M.R., de-Lucchi C., Medina A., Martinez-Valverde A., Uauy R., Gil A. (1988). Changes in the fatty acids pattern of red blood cell phospholipids indduced by type of milk, dietary nucleotide supplementation, and postnatal age in preterm infants. J. Pediatr. Gastroenterol. Nutr..

[24] Hess J.R., Greenberg N.A. (2012). The role of nucleotides in the immune and gastrointestinal systems: Potential clinical applications. Nutr. Clin. Pract..

[25] Gil A. (2002). Modulation of the immune response mediated by dietary nucleotides. Eur. J. Clin. Nutr..

[26] Carver J.D., Pimentel B., Cox W.I., Barness L.A. (1991). Dietary nucleotides effects upon immune function in infants. Pediatrics.

[27] Broberg S., Sahlin K. (1989). Adenine nucleotide degradation in human skeletal muscle during prolonged exercise. J. Appl. Physiol..

[28] Novak D.A., Carver J.D., Barness L.A. (1994). Dietary nucleotides affect hepatic growth and composition in weanling mouse. J. Parenter. Enteral Nutr..

[29] Jimenez J., Boza J., Suarez M.D., Gil A. (1992). Changes in fatty acid profiles of red blood cell membranes mediated by dietary nucleotides in weanling rats. J. Pediatr. Gastroenterol. Nutr..

[30] Carver J.D. (1994). Dietary nucleotides: Cellular immune, intestinal and hepatic system effects. J. Nutr..

[31] Ogita K., Suita S., Taguchi T., Yamanouchi T., Masumoto K., Nakao M. (2002). Roles of nucleosides and nucleotidemixture in small bowel transplantation. Nutrition.

[32] Scolpesi F., Verkeste C.M., Paola D., Gazzolo D., Pronzato M.A., Bruschettini P.L., Marinari U.M. (1999). Dietary nucleotide supplementation raises erythrocyte 2,3-diphosphorglycerate concentration in neonatal rats. J. Nutr..

[33] Joyner M.J., Coyle E.F. (2008). Enduranceexerciseperformance: The physiology of champions. J. Physiol..

[34] Armstrong L.E., Casa D.J., Millard-Stafford M., Moran D.S., Pyne S.W., Roberts W.O. (2007). American college of sports medicine position stand. Exertional heat illness during training and competition. Med. Sci. Sports Exerc..

[35] Huang C.F., Wang C.C., Wu T.C., Chu C.H., Peng H.J. (2007). Effect of sublingual administration with a native or denaturated protein allergen and adjuvant CpG oligodeoxynucleotides or cholera toxin on syzstematic T(H)2 immune responses and mucosal immunity in mice. Ann. Allergy Asthma Immunol..

